# Cowpea (*Vigna unguiculata* L.) production, genetic resources and strategic breeding priorities for sustainable food security: a review

**DOI:** 10.3389/fpls.2025.1562142

**Published:** 2025-07-29

**Authors:** Dong-Kwan Kim, Kingsley Ochar, Kanivalan Iwar, Bo-Keun Ha, Seong-Hoon Kim

**Affiliations:** ^1^ Resource Management Division, Jeollanamdo Agricultural Research and Extension Services, Naju, Republic of Korea; ^2^ Council for Scientific and Industrial Research, Plant Genetic Resources Research Institute, Bunso, Ghana; ^3^ National Agrobiodiversity Center, National Institute of Agricultural Sciences, Rural Development Administration, Jeonju, Republic of Korea; ^4^ Department of Applied Plant Science, Chonnam National University, Gwangju, Republic of Korea

**Keywords:** cowpea, germplasm conservation status, OMICs, research and breeding development, strategic breeding priorities, trend of production

## Abstract

Cowpea is one of the most important staple crops, known for food security and agricultural sustainability. The crop has a multifaceted use, including food, feed, and soil fertility management. It is a staple food, especially in Africa and parts of Asia and the Americas, where it is valued for its rich nutritional content. Though cowpea is a climate resilient legume, the extent of its environmental adaptability and its utility value vary considerably across different production regions. Each region has unique conditions, including varying climates, soil types, and pest pressures. Given this versatile and diverse nature of cultivation environments, different varieties are needed to address all regional challenges. Breeding efforts often prioritize producing a range of varieties with different plant types, growth habits, and maturity periods in order to maximize yields and enhance environmental resilience, while also addressing region-specific needs. Meanwhile, genetic diversity among crop resources is essential for effective breeding, as targeted strategic breeding can significantly improve crop productivity, enhance market value, and better meet consumer preferences. With the aim to further strengthen research efforts in cowpea breeding for increased yield productivity for food security, this review examined recent global cowpea production, state of germplasm resources conservation and breeding priorities across different regions. Based on the current knowledge, further progress is required in addressing specific regional challenges, such as enhancing pest resistance, drought tolerance, and nutritional quality to ensure continued contribution of cowpea to global food security and agricultural sustainability.

## Graphical Abstract

Genetic resources coupled with strategic breeding priorities are requisite to increase cowpea production, improve market value and to meet consumer demands.

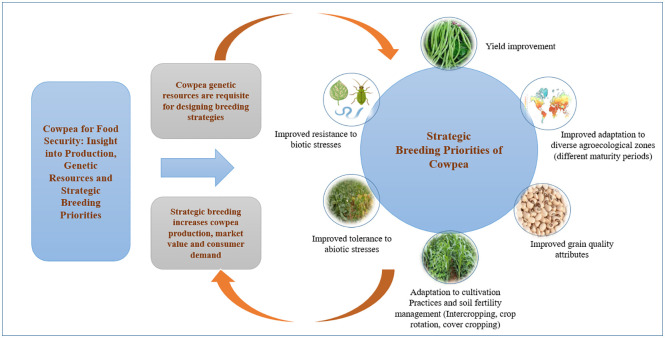

## 1 Introduction

In an era marked by the burgeoning global human population, diminishing arable lands and mounting impacts of climate change, efforts to achieve food security has become a pressing global concern (Pradhan et al., 2018; Omomowo and Babalola, 2021; Popescu et al., 2024). By 2050, the global population is projected to reach ~10 billion, which will likely intensify the strain on already overburdened agricultural systems and the need for a substantial increase in food production to meet the growing demand (Dagunga et al., 2023; O’Sullivan, 2023). Besides, as climate change intensifies, unpredictable weather pattern will continue to disrupt global agricultural systems, increase crop vulnerability to environmental stresses, and consequently, crop yield penalties (Ochar et al., 2022; Rosegrant et al., 2024). These disruptions are threats to food supply stability, and capable of aggravating hunger emergencies and malnutrition in vulnerable communities worldwide (Gomes et al., 2024; Ogwu et al., 2024). Thus, this necessitates innovative solutions, including strategic crop breeding, investing in research for climate-resilient crop development, and intensifying international collaboration on sustainable food production models (McClements et al., 2021; Usigbe et al., 2024). Cowpea is an annual warm season legume, widely cultivated and embraced for its agricultural resilience and nutritional sustenance (Muñoz-Amatriaín et al., 2017). Its historical roots, in terms of origin and domestication are traced to Africa, where it has served as a dietary staple for centuries (Panzeri et al., 2022; Wu et al., 2024). The crop ranks among the most important list of global legumes due to its exceptional adaptability to diverse agroecosystems, and relative contribution to global legume production (Semba et al., 2021; Chepngeno et al., 2024). As a legume, cowpea possesses the ability to fix atmospheric nitrogen, enriching soil fertility, even under suboptimal conditions (Mekonnen et al., 2022; Becker et al., 2024). This resilience is particularly crucial in regions where smallholder farmers rely heavily on cowpea cultivation for income and dietary protein (Seo et al., 2020; Faye et al., 2024).

Now it is clear that persistent change of climatic variables, characterized by erratic rainfall patterns, prolonged droughts, and increased incidences of pests and diseases pose daunting challenges to global agricultural productivity. However, cowpea exhibits a notable resilience to environmental challenges, with inherent attributes that distinguishes it as a climate-smart crop, especially, when compared to several other legumes (Gong et al., 2023). Yet, the extent of adaptability and utilization value of cowpea and, thus the reasons for its cultivation vary across regions, indicating the need for region-specific breeding strategies to improve its performance in the face of shifting climatic conditions (Horn and Shimelis, 2020; Asiwe, 2022; Mohammed et al., 2024). Generally, breeding targets of cowpea include the development of high-yielding varieties, with improved grain quality, and enhanced resilience to both biotic and abiotic stresses (Gomes et al., 2019; Mekonnen et al., 2022). These efforts are especially relevant as cowpea, though, its production is primarily concentrated in Africa and Asia, is gaining increasing recognition in other regions. This growing interest stems not only from the potential of the crop to strengthen food systems and nutritional security, but also by its valuable role in enhancing soil fertility within cropping systems (Abebe and Alemayehu, 2022; Affrifah et al., 2022). While cowpea cultivation in the Americas and Europe are relatively nascent compared to its extensive history in Africa, there are a growing number of breeding initiatives that are ongoing across different regions. These initiatives, in terms of priority, may differ significantly or overlap across different regions and nations, reflecting the diverse agricultural needs and climatic conditions. The regional differences in cowpea end use highlights the need for context-specific breeding strategies, aimed at maximizing the crop’s potential to address local agricultural challenges effectively. Therefore, the ultimate aim of this review is to examine the current state of global cowpea production, the state of genetic resources of the crop and discuss breeding priorities for enhanced yield productivity and resilience. By understanding these regional approaches, we can better appreciate the comprehensive efforts to leverage the potential of cowpea as a crucial component of global food security and agricultural sustainability.

## 2 The role of cowpea in global food security, rural economies, and agricultural sustainability

Cowpea provides a reliable source of nutrition for millions of people across the globe, especially as essential supplement to animal protein. The grains are packed with proteins, dietary fiber, vitamins, and minerals essential in addressing nutritional deficiencies (Wu et al., 2024). Across sub-Saharan Africa and parts of Asia, where cowpea is very much produced, the crop plays a pivotal role in traditional farming systems, where the grains contribute significantly to household food security and income generation (Da Silva et al., 2018; Sarr et al., 2021). Da Silva et al. emphasized that cowpea is both a staple food and a nutraceutical, contributing to health and wellness through its high nutritional content (Da Silva et al., 2018). Their study revealed that cowpea consumption helps combat malnutrition and micronutrient deficiencies, particularly in low-income communities where access to diverse foods is limited. Omomowo and Babalola also noted that the resilience of cowpea to harsh environmental conditions, such as drought and heat, makes it an ideal crop for food security in regions affected by climate change (Omomowo and Babalola, 2021). Their study highlighted further the ability of the crop to thrive in arid areas, and contribute to stability of food production systems where other crops might fail. This climate resilience is a crucial factor in maintaining food security in regions vulnerable to environmental shocks. Cowpea is also a cornerstone of rural economies, particularly in sub-Saharan Africa, where it is a major cash crop for smallholder farmers. Highlighting the economic importance of cowpea in Nigeria, Nwagboso et al. noted the potential of cowpea to drive agricultural transformation and poverty reduction (Nwagboso et al., 2024). They pointed out that cowpea is not only a source of food, but also an important income-generating crop for farmers, particularly in the dry season. This economic impact is evident in the increased market participation and income generation associated with cowpea cultivation as documented by Manda et al., who found that cowpea producers in northern Nigeria reported significant improvements in household food security and income (Manda et al., 2020). Kebede and Bekeko also emphasized the economic significance of cowpea in Ethiopia, where it serves as a major crop for both local consumption and export (Kebede and Bekeko, 2020). The income generated from cowpea farming allows rural households to invest in other aspects of their livelihoods, such as education and healthcare, further contributing to poverty alleviation. The ability of the crop to grow with minimal water requirements makes it an attractive option for sustainable farming in areas facing water scarcity, thereby promoting agricultural sustainability. Fatokun discussed the challenges of enhancing sustainable cowpea production but also highlighted the inherent potential of the crop to withstand drought and its role in agroecological systems (Fatokun et al., 2002). This characteristic is becoming increasingly important as climate change exacerbates water shortages in many agricultural regions. Despite its potential, cowpea production faces several challenges, including both biotic and abiotic stresses, low productivity, and limited access to high-quality seeds (Nwagboso et al., 2024). The study by Gomes et al. has shown that breeding efforts, including development of high-yielding, pest-resistant varieties are critical to addressing these challenges (Gomes et al., 2019). Their research suggests that improvements in cowpea breeding could significantly enhance food security and income generation in Africa. The introduction of improved varieties with resistance to biotic stresses like aphids and root rot disease is expected to contribute to more sustainable and resilient production systems. Moreover, the economic and nutritional benefits of cowpea are often hampered by poor post-harvest management, limited processing infrastructure, and under-developed markets. So in addition to breeding, Medendorp et al. discussed the need for enhanced value chains, including improved storage, processing, and marketing, to maximize the economic potential of cowpea and reduce post-harvest losses (Medendorp et al., 2022). Strengthening these value chains could open new markets and improve the profitability of cowpea farming.

## 3 Current status of global cowpea production, harvest area and yield

Cowpea is a multipurpose crop, serving as food, feed, and for soil fertility and conservation management (Horn and Shimelis, 2020; Omomowo and Babalola, 2021). Cultivated globally across diverse agroecological zones, cowpea is resilient to heat, drought, and low soil fertility, typical characteristics that enables it to flourish in regions with unpredictable rainfall, and poor agricultural conditions (Moreira et al., 2023). This adaptability potential, combined with nitrogen-fixing ability makes cowpea a crucial crop for cultivation in semi-arid areas, characterized by heavy reliance on rain-fed agriculture (Tankari et al., 2021). While many smallholder farmers in the tropics and semi-tropics grow cowpea to generate incomes for their livelihoods, research institutions such as International Institute of Tropical Agricultural (IITA) may embark on large scale cultivation for seed multiplication purpose (Ogunsola et al., 2023). The total harvested area of cowpea for its dry grains is estimated to cover over 15 million hectares worldwide, yielding around 9 million metric tons (Olorunwa et al., 2023; Laosatit et al., 2024). The growing recognition of the potential of cowpea as a climate-resilient crop highlights its role in enhancing food security and nutrition (Lonardi et al., 2019). Globally, dry grain production, harvested area, and yield of cowpea have steadily increased over the past years (https://www.fao.org/faostat/en/; Accessed 2024/07/17). While production is widely distributed, the largest proportion (95.4%) of global dry grain cowpea is produced in Africa, particularly West Africa, followed by Asia (2.9%), with the Americas (1.3%) and Europe (0.5%) recording smaller shares.

### 3.1 Trend in cowpea production, harvest area and yield in Africa

**Figure 1:**
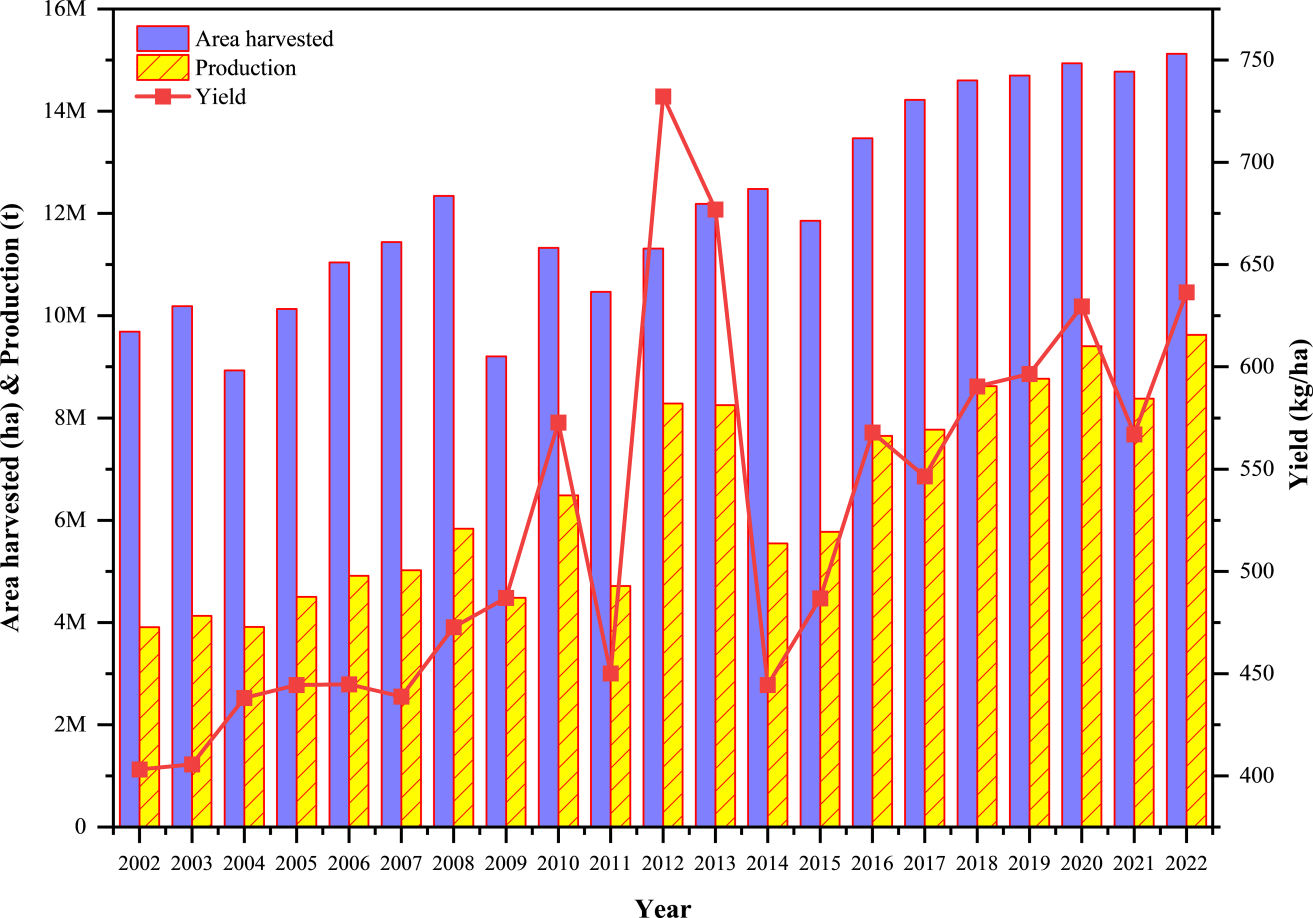
Trend in grain cowpea production, harvest area, and yield over the past two decades in Africa (adapted from https://www.fao.org/faostat/en/; Accessed 2024/07/17). Production, harvested area, and yield of dry grain cowpea reveals a rising trend over the past years, with the highest increases recorded in 2012 and 2013.

In Africa and across the globe, Nigeria has over the years maintained a constant position as the leading global producer of grain cowpea, exceeding 4 million hectares of planting area (**Figure 1**; **Table 1**), and a total production volume of over 4 million metric tonnes annually (Laosatit et al., 2024). Other countries in the continent which produce significant quantities of cowpea include Niger, Burkina Faso, Ghana and Mali, all in Western Africa (**Table 1**). In Sub-Saharan Africa, cowpea production is typically carried out by smallholder farmers, often as sole cropping or an intercrop with crops such as, maize, sorghum, or millet (Saka et al., 2019; Ogunsola et al., 2023). The primary objective of cowpea growers in Africa is to produce dry grains for domestic consumption (Tijjani et al., 2015). Thus, dry grains of cowpea are one of the dominant grain legumes traded in local markets across many African countries and serves as a cash-generating commodity for farmers and small to medium-sized entrepreneurs (Moussa et al., 2011; Occelli et al., 2024). Additionally, cowpea haulms provide important fodder for ruminants, especially in the Sahel regions of West and Central Africa (Boukar et al., 2016, Boukar et al., 2020). Over the past two decades, production, harvested area, and yield of grain cowpea have generally exhibited an upward trend, with the most notable yield recorded in 2012 and 2013, which correlated positively with increased production (**Figure 1**). The enormous increase in cowpea yield per hectare since 2002 can largely be accounted for by several key developments, including adoption of improved cowpea varieties with multiple genetic traits of resistance to pests and diseases, and drought (Boukar et al., 2019a). Advances in breeding technologies like marker-assisted selection (MAS) and genomic selection have also facilitated the release of better cultivars (Muñoz-Amatriaín et al., 2017). Additionally, agronomic practices like planting density optimization, integrated pest management (IPM), and soil fertility management have all collectively contributed towards improved productivity of the crop within the region (Herniter et al., 2019). However, it must be admitted that this positive trajectory appears not to be uniform across all cowpea-producing areas within the region. While some countries such as Nigeria, Niger, Burkina Faso, Ghana might have experienced notable improvements in yield, largely due to targeted breeding programs and better dissemination of improved varieties, other countries might have seen stagnant or marginal increases in yield per hectare, despite increases in cultivated area. This regional disparity underscores existing breeding and dissemination gaps, particularly in tailoring varieties to local agroecological conditions, biotic stressors, and farmer preferences. For regions with stagnant yields, this may be due to several constraints such as limited access to improved varieties, weak seed systems, insufficient breeding focus on regional abiotic stressors (e.g., soil acidity, heat stress), and emerging pest pressures such as *Aphis craccivora* and *Maruca vitrata, Callosobruchus maculatus* Fab, for which the varieties may have partial or no resistance (Nboyine et al., 2024a, Nboyine et al., 2024b; Addae et al., 2020). Additionally, the adoption of improved cowpea lines in some regions might be hindered by low extension support, limited farmer awareness, and poor integration of gender-responsive breeding approaches (Jinbaani et al., 2023; Horn and Shimelis, 2020). With continued investment in new technology, such as precision agriculture, intensified integration of OMICs technologies into breeding programs, and adoption of climate-smart agriculture practices, cowpea yield will continue to increase (Lonardi et al., 2019). Projected advancements in gene editing technologies, including CRISPR/Cas9, and enhanced biofortification methods may potentially enable yield improvements of some 20-30% or more within the next decade, provided that these technologies are effectively integrated into breeding and farming practices (Muñoz-Amatriaín et al., 2017). For instance, the release of the first-ever Pod Borer Resistant (PBR) cowpea in Ghana in 2024 represents a major milestone that is expected to further boost cowpea productivity by minimizing losses from *Maruca vitrata* infestation, one of the most destructive pests of cowpea Fab (Nboyine et al., 2024a) (https://thebftonline.com/2024/07/26/breakthrough-first-ever-pod-borer-resistant-cowpea-unveilled/). A recent report by the CSIR-Savannah Agricultural Research Institute revealed the development of cowpea lines resistant to multiple insect pests, emphasizing the role of biological control methods in sustainable pest management systems across West Africa (Togola et al., 2023). Furthermore, new breeding techniques that leverage tissue culture are overcoming cross-incompatibility barriers between domesticated and wild cowpea relatives, allowing the introgression of novel traits such as stress tolerance and enhanced nutritional profiles that were previously difficult to incorporate into cultivated varieties (Vacu et al., 2025). These advances, when combined with ongoing improvements in breeding technologies like marker-assisted selection and genomic selection, along with the integration of OMICs technologies and precision agriculture, provide a strong foundation for achieving even greater gains in cowpea yield and resilience in the coming decade. However, despite these promising developments, limited access to OMICs tools such as genomics, transcriptomics, proteomics, and metabolomics in many regions, particularly in sub-Saharan Africa may pose a barrier to widespread application. Furthermore, weaknesses in seed systems, including poor distribution networks, limited availability of improved seeds, and insufficient farmer education may constrain the adoption of new cultivars. Regulatory bottlenecks, such as delays in the approval and release of genetically improved varieties, coupled with strict biosafety regulations can hinder the rapid deployment of innovative technologies. Addressing these challenges through investment in research infrastructure, strengthening seed systems, and streamlining regulatory frameworks will be crucial to fully realize the potential of current scientific advances in cowpea improvement.

**Table 1:** Top 20 global cowpea producing countries, excluding Brazil.

S/N	Country	Production in tones (million)	Area harvested in hectares (million)	Yield per hectare (in thousands)
–	World	9.775	15.190	6.435
1	Nigeria	4.134	4.776	8.654
2	Niger	2.866	5.851	4.898
3	Burkina Faso	0.829	1.719	4.825
4	Ghana	0.320	0.192	16.625
5	Mali	0.250	0.492	5.083
6	Sudan	0.184	0.292	6.283
7	Cameroon	0.182	0.222	8.177
8	Senegal	0.152	0.294	5.180
9	United Republic of Tanzania	0.149	0.144	10.308
10	Kenya	0.131	0.223	5.882
11	Myanmar	0.107	0.118	9.113
12	Mozambique	0.083	0.344	2.405
13	Democratic Republic of the Congo	0.082	0.184	4.449
14	Yemen	0.081	0.031	26.102
15	Malawi	0.050	0.102	4.881
16	Haiti	0.030	0.041	7.272
17	Madagascar	0.022	0.023	9.376
18	Peru	0.019	0.014	13.526
19	Serbia	0.016	0.004	33.726
20	Sri Lanka	0.015	0.014	10.353

### 3.2 Trend in cowpea production, harvest area and yield outside Africa

**Figure 2:**
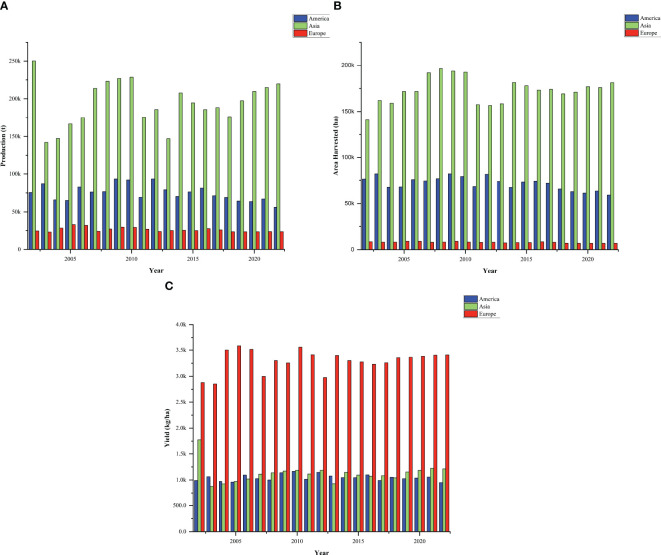
Trend in grain cowpea production **(A)**, harvest area **(B)**, and yield **(C)** over the past two decades in Asia, Americas and Europe (adapted from https://www.fao.org/faostat/en/; Accessed 2024/07/17). Generally, a clear contrast in yield trajectories is observed across cowpea-growing regions over the past two decades. Grain cowpea production, and harvested area in Asia shows a fluctuating trend over the past two decades, but a consistent upward trend in recent years. Within the past two decades, grain cowpea production and harvest area in the Americas indicate a consistent downward trend, as production for grains decline. Compared with previous years, grain cowpea production in Europe also portrays a downward trend in recent years.

Outside Africa, cowpea is cultivated in Asia, the Americas, and parts of Europe (Muñoz-Amatriaín et al., 2017; Liang et al., 2024). Contrast to dry grains which are popular in Africa, in many parts of Asia, the leaves, fresh grains, and green pods of asparagus bean (or yard-long bean) are primarily consumed as vegetables (Owade et al., 2020; Pasquet et al., 2021; Mekonnen et al., 2022). Asparagus bean has a characteristic climbing and twining growth habit and elongated pods, contrast to the erect or semi-erect growth habit of grain cowpea (Seo et al., 2020). Nonetheless, Myanmar, Yemen, Sri Lanka, China, Philippines (https://www.fao.org/faostat/en/; Accessed 2024/07/17) and India produce substantial quantities of dry cowpea. In China, cowpea is primarily cultivated in the mountainous areas, either as a monoculture in terraced fields and gardens or intercropped with other crops, with cultivation area and total yield steadily increasing from previous years (Gong et al., 2023). For the Republic of Korea, cowpea is still a minor crop in terms of production and consumption volume (Seo et al., 2020). However, in recent years, cowpea research is gaining momentum as part of efforts to diversify crop options and enhance agricultural sustainability. Overall, production, and harvested area of grain cowpea in Asia reveals fluctuations in values over the past two decades, but a consistent upward trend is observed in recent years. (**Figure 2A**), perhaps attributed to improved awareness of the suitability of the crop in climate-resilient farming systems, particularly in semi-arid and marginal environments. Cowpea production and harvested area in Asia have exhibited fluctuating patterns over the past two decades. This expansion in harvested area reflects a growing recognition of agronomic and economic value of cowpea, particularly in drought-prone regions where the crop’s resilience and short growth cycle make it a favorable option for smallholder farmers. Despite this progress or positive trends in harvested area, yield per hectare has remained relatively stagnant in many cowpea growing areas in Asia, indicating that increased production is more a result of expanded cultivation area than substantial yield improvements. This yield stagnation highlights underlying breeding and dissemination challenges. There is a growing need to intensify region-specific cowpea breeding strategies that prioritize high-yielding, pest-resistant, and early-maturing lines suited to diverse Asian agroecologies. Strengthening research collaboration, particularly with African institutions like IITA and CSIR-Savannah Agricultural Research Institute in Ghana could accelerate genetic gain in Asian cowpea breeding efforts. Addressing these breeding gaps is essential to lifting yield ceilings and ensuring that recent production growth translates into long-term productivity and food security gains in the region.

In the United States, cowpea is extensively cultivated as a multipurpose crop for forage, green manure and cover cropping (Horn and Shimelis, 2020; Pasquet et al., 2021). Cowpea production in USA is highly concentrated in California and Midwestern and southern states, with the dry grains used as staple in the southern states (Osipitan et al., 2021). Despite these uses, grain yield levels in the U.S. have remained relatively static over the past two decades. This stagnation can be attributed to the limited breeding focus on improving grain-specific traits, such as seed size, cooking quality, and drought tolerance, due to the prioritization of cowpea as a cover crop over its potential as a food legume. As a result, there is a noticeable gap between the yield potential of U.S. cowpea germplasm and that of newly improved African varieties bred for grain productivity. In South America, significant quantities are produced in Haiti, and Peru (https://www.fao.org/faostat/en/; Accessed 2024/07/17), where it contributes to local food security. Based on 2022 production statistics, Haiti produced the largest quantity of cowpea in this region, with annual production value of 29933.17 tonnes, on ~41161.00 hectares of land. Other leading producers in the region include Trinidad and Tobago, Jamaica, and Guyana, where cowpea cultivation contributes significantly to regional food security and agricultural diversity (Pasquet et al., 2021). In Brazil, where cowpea production was originally dominated by small and medium farmers in the North and Northeast is now increasingly been adopted by large scale producers using advanced technologies (Barbosa et al., 2016). Nonetheless, over the past two decades, the trend of production and harvest area in the Americas indicates a consistent downward trend, suggesting a decline in production for grains (**Figure 2B**). While countries like Brazil have made strides in cowpea research through EMBRAPA (Brazilian Agricultural Research Corporation), the broader Latin American region may still face breeding limitations, including underdeveloped local breeding programs, inadequate investment in legume research, and limited regionally adapted varieties. These factors may contribute to stagnation or decline in yields. This emphasizes a critical need for reinvigorated cowpea breeding efforts across the region, with greater emphasis on dual-purpose varieties that meet both agronomic and food-use requirements. Incorporating modern molecular tools, strengthening regional breeding programs, and aligning varietal development with end-user preferences such as grain quality, maturity period, and pest resistance would help close existing yield gaps and better harness the potential of cowpea in the regions.

Legumes are also a central component of the Mediterranean diet, and processed foods in Europe (Cusworth et al., 2021). This has led to a surge in demand for plant-based proteins, both for food and feed, particularly, following the shift toward organic, and non-GMO products (Rubiales et al., 2021). Over the years, the climate-resilience potential of cowpea has gained significant recognition in Europe, accounting for around 0.3% of pulses produced (Carvalho et al., 2017). Major cultivation areas are concentrated in the Southern Mediterranean region including Greece, Italy, Spain, Cyprus, Croatia, Portugal and Serbia, Bosnia and Herzegovina, and North Macedonia, where the crop is valued for its adaptability to local climates (Lazaridi and Bebeli, 2023). It is grown mainly for its nutritional benefits, ability to thrive in arid conditions and for improving soil health through nitrogen fixation. Serbia produces significant amount of cowpea, ranking among the top 20 global cowpea producers (**Table 1**). However, relative to the values in previous years, recent cowpea production in Europe shows a downward trend (**Figure 2C**), perhaps due to factors such as changing climatic conditions, reduced agricultural land allocated to legume production, pest and disease pressures, and a decline in farmer interest or knowledge regarding cowpea cultivation. Similarly, harvest area in Europe has gradually decreased over time, possibly due to limited emphasis on grain legumes and reduced suitability or profitability in local cropping systems. Generally, over the past two decades (2002–2022), notable differences have been observed in the trends of grain cowpea production (tonnes), harvest area (hectares), and yield (kg/ha) across Asia, the Americas, and Europe. In terms of total production and harvest area, Asia consistently recorded the highest values, followed by the Americas, with Europe trailing behind. Particularly for the past half a decade, cowpea production continues to rise in Asia, while both the Americas and Europe have experienced a declining trend. Asia shows the largest area under cowpea cultivation, reflecting the crop’s dietary importance in the region. Conversely, the Americas show a declining pattern in harvested area, suggesting a potential shift in agricultural priorities or land use. Interestingly, despite the relatively smaller harvest area and lower total production, Europe recorded the highest yields (kg/ha) among the three regions. Asia followed, while the Americas had the lowest overall yields. This suggests that cowpea cultivation in Europe may prioritize producing dry grains for consumption, rather than other end-use priorities such as cultivation for soil management (Osipitan et al., 2021).

## 4 Strategic breeding priorities of cowpea

Given the versatility nature and diversity of cultivation environments of cowpea, a fewer number of varieties cannot meet all the diverse conditions and requirements (Singh, 2016). Each growing region has unique challenges such as varying climates, soil types, and pest pressures that a one-size-fits-all approach cannot address. Besides, cowpea growers and consumers often exhibit preferences for specific traits as highlighted in the study by Asiwe (Asiwe, 2022). Horn and Shimelis further noted that the agronomic needs for cowpea vary across different regions (Horn and Shimelis, 2020). As a result, it is essential to develop multiple varieties adapted to specific conditions to ensure optimal performance. Breeding must produce various varieties with different plant types, growth habits, and maturity to enhance yield and resistance to biotic and abiotic stresses while meeting regional needs. The primary goals in cowpea breeding are to enhance grain yield and quality while addressing biotic and abiotic stresses (Pan et al., 2017). Among others, strategic breeding priorities of cowpea are highlighted in subsequent subsections (4.2.1 - 4.2.6). Generally, whereas diversity of genetic resources of the crop is requisite for breeding, strategic breeding priorities increases crop productivity, enhances market value, consequently, promote food security.

### 4.1 Maturity time for diverse agroecologies

Developing early, extra early and medium maturing cowpea varieties is an important objective of the crop, with a focus on developing varieties that mature quickly, and suitable for regions with short growing seasons or specific niche cropping systems (Singh, 2016). These varieties enable farmers to maximize their production in areas with limited rainfall or short growing periods by allowing for faster harvests (Huynh et al., 2018). Generally, in terms of pod maturity, cowpea varieties can be classified into extra-early, early and late maturing, corresponding with pod maturity days <60, 61–80 and >80 from sowing respectively (Dorvlo et al., 2022). In another study, Asiwe, who described cowpea maturity using early (70–90 days), medium (91–100 days and late-maturing (101–120 days) revealed that early-maturing varieties are suitable for double cropping in high rainfall areas (Asiwe, 2022). Medium and late varieties have dual-purpose use, production of dry grains and fodder, though these types of cowpea usually produce less seeds. Cowpea breeding programs may aim at developing early and medium maturity lines, with good grain quality suitable for both sole cropping and intercropping systems. Additionally, quick maturation can facilitate multiple cropping cycles within a single year, increasing overall farm productivity. Varieties with medium-maturity periods are suitable for sole cropping, intercropping, and mixed farming systems (Singh, 2016). Generally, breeding varieties for different maturity time increases the crop’s adaptation and scope for cultivation in different agroecological zones (Ogunsola et al., 2023).

### 4.2 Tolerance to abiotic stresses

Under extreme conditions, yield and productivity of cowpea can be vulnerable to drought, heat, salinity, flooding, and soil nutrient, such as low soil phosphorus content. Improved tolerance and adaptation to abiotic conditions increases the resilience and productivity of cowpea. Therefore, cowpea breeding efforts prioritize varieties that can tolerate drought, withstand high temperatures, and thrive in soils with low nutrient levels. In a previous study, Hall highlighted the need for developing varieties that are water-use efficient, heat tolerant, and having deeper root system to increase response to drought (Hall, 2012). Erratic rainfall patterns that characterize the semi-arid tropics, with irregular starts and early cessation calls for resilient varieties to enhance the drought tolerance of the crop (Dorvlo et al., 2022). Early maturing varieties are noted to be efficient in water use, and are particularly suitable for cultivation in drought-prone areas, as plants escape terminal droughts (Horn and Shimelis, 2020).

### 4.3 Resistance to biotic stresses

Ensuring ecological sustainability in crop production is highly important, and calls for reduced application of chemical for controlling pests, diseases, parasitic weeds and nematodes. Crop varieties with resistance to biotic factors help promote sustainable agriculture and ecology. Breeding prioritize resilience to prevalent diseases, insect pests, parasitic weeds and nematodes commonly associated with crops (Singh, 2016). Enhanced cowpea resistance ensures higher and more stable yields. In a recent study, Asiwe developed and registered ten high grain yield cowpea genotypes, expressing resistance to both pests and pathogens, valuable for maximizing yield and profit by farmers (Asiwe, 2022). The plant parasitic weed, *Striga gesnerioides* can pose severe constraints in cowpea production, causing yield losses ranging between 80 and 100% (Omoigui et al., 2017). In genetic enhancement study, Omoigui et al. used marker-assisted selection strategy to incorporate *Striga* resistance from IT97K-499–35 into the farmer-preferred cowpea cultivar ‘Borno Brown’ for Nigeria’s savannas (Omoigui et al., 2017). Out of 47 BC1F2 populations, 28 lines with desirable traits, including *Striga* resistance and preferred seed characteristics were identified and tested. Aphids are one of the most notorious pests and pathogen vectors of cowpea, and breeding resistant varieties has been an effective technique to sustain yield productivity of the crop (Mofokeng and Gerrano, 2021). Over the last few years, there has been vast improvement in the application of biotechnology tools towards the improvement of cowpea, especially in the production and post-harvest constraints through transgenic means and in the generation of genomic resources. The cowpea bruchid (*Callosobruchus maculatus*) is one of the most significant post-harvest pests of cowpea storage that causes severe grain loss in the tropics (Amusa et al., 2018). A recent study published promising field results of genetically modified cowpea lines expressing a bean α-amylase inhibitor 1 (αAI-1) (Nboyine et al., 2024b). The transgenic lines exhibited high-level resistance to bruchid infestation in both confined field trials and controlled storage conditions, with up to 98% inhibition of adult emergence compared to non-transgenic controls. Remarkably, these lines exhibited normal agronomic performance and seed viability, indicating the stability and utility of the trait in breeding programs for post-harvest losses. This is a crucial step toward the inclusion of biotechnological solutions for cowpea storage tolerance.

### 4.4 Enhanced yield

Breeding cowpea for enhanced yield is a critical objective in an effort to meet growing food demands and improve agricultural sustainability (Manu et al., 2024). By targeting yield, breeding programs have focused on both grain cowpea and vegetable cowpea, targeting key yield component traits such as pod number, seed size, plant architecture, earliness, and pest and disease resistance (Metwally et al., 2021; Asiwe, 2022). These traits are integral to optimizing yield under various environmental conditions. At the IITA, significant progress has been made in developing high-yielding, stress-resistant varieties, using conventional, marker-assisted selection (MAS), and hybridization techniques to enhance yield traits (Mahesha et al., 2022). For instance, the development of the variety IT89KD-288 is an outcome of breeding efforts targeting early maturity, drought tolerance, and high grain yield (Chamarthi et al., 2019).

### 4.5 Improved grain quality

Grain quality, along with yield is a principal breeding goal in cowpea (Singh, 2016), involving breeding varieties with seeds that are not only nutritionally rich in protein, and essential micronutrients, but also containing beneficial health compounds. High-protein and nutrient-dense seeds contribute to better human nutrition and health. Additionally, such seeds can enhance the overall quality and market value of the crop, benefiting both producers and consumers. In their study, Ogunsola et al. observed specific cowpea characteristics such as sweetness, seed coat color, seed size, and cooking value that significantly influences farmers’ selection of cultivars for commercial cultivation (Ogunsola et al., 2023). Similar observation was also highlighted by Gondwe et al. who noted that seed coat color, texture, and size impact consumer preferences and demand (Gondwe et al., 2019). Considerable studies on protein and minerals content of cowpea germplasm have been conducted, with desirable resources identified which are useful for breeding nutrient dense or biofortified cowpea varieties (Santos and Boiteux, 2015; Gerrano et al., 2019; Duraipandian et al., 2022). In recent years, cowpea breeders have considered developing next-generation biofortified varieties to combat micronutrient deficiencies. They achieve this by exploring and exploiting the substantial genetic variability in nutritional components to enhance genetic biofortification (Dhanasekar et al., 2021). Exploring diversity of genetic resources, incorporating modern breeding methods, and utilizing innovative techniques, such as gene editing and multi-omics analyses could accelerate progress in breeding biofortified cowpea (Sodedji et al., 2024).

### 4.6 Soil fertility management

Changes in land use and improper management practices normally deplete agricultural lands of soil nutrient, negatively impacting soil biochemical and microbial indicators, and causing low available soil nutrients needed for optimum crop growth and grain yields (Ruiz-de-Cenzano et al., 2015; Mganga et al., 2016). As a legume crop, cowpea has a symbiotic nitrogen fixation ability, essential for soil nutrient management and promoting ecological sustainability (Rubiales et al., 2021). Kulkarni et al. noted that cowpea, along with other legumes, is a key player in sustainable agricultural systems, especially in regions where soil degradation is a major challenge (Kulkarni et al., 2018). Their study emphasized the importance of legumes like cowpea in enhancing soil health and maintaining long-term agricultural productivity. In their study, Jacobsen et al. suggested that cereal crop production can be well sustained, especially in rain-fed dry areas, using grain legumes as soil fertility improvement strategy (Jacobsen et al., 2012). The crop holds tremendous prospects in enhancing cereal grain yields when integrated into field crop cultivation systems, either in the form of rotation or intercropping (Périnelle et al., 2021; Roohi et al., 2022). In Africa for instance, many farmers often desire to have cereals and legumes produced on the same land, and undertake cowpea cultivation as an intercrop in cereal fields. To achieve the goal of soil fertility management, super nodulation varieties are essential for integration into legume-cereal cropping systems (Namatsheve et al., 2021). Breeding varieties with less dense canopy, and higher in nitrogen-fixing capabilities is essential for sustainable agricultural practices. Suitability for alternate row and double row cereal-cowpea intercropping was one of the key targets in the works of Asiwe (Asiwe, 2022), who developed cowpea varieties with characteristic narrow leaf blades for high density monocropping and intercropping. In another study, Osipitan et al. found that cowpea production in the U.S.A is geared towards soil management and livestock feed, with substantial proportion of land dedicated to soil management (Osipitan et al., 2021). Rotational cropping of cowpea with crops such as sorghum, corn, wheat, and cotton helps improve soil fertility, by reducing pest buildup, and enhance yields in subsequent planting cycles (Kumari et al., 2024).

## 5 Global cowpea germplasm conservation status

**Figure 3:**
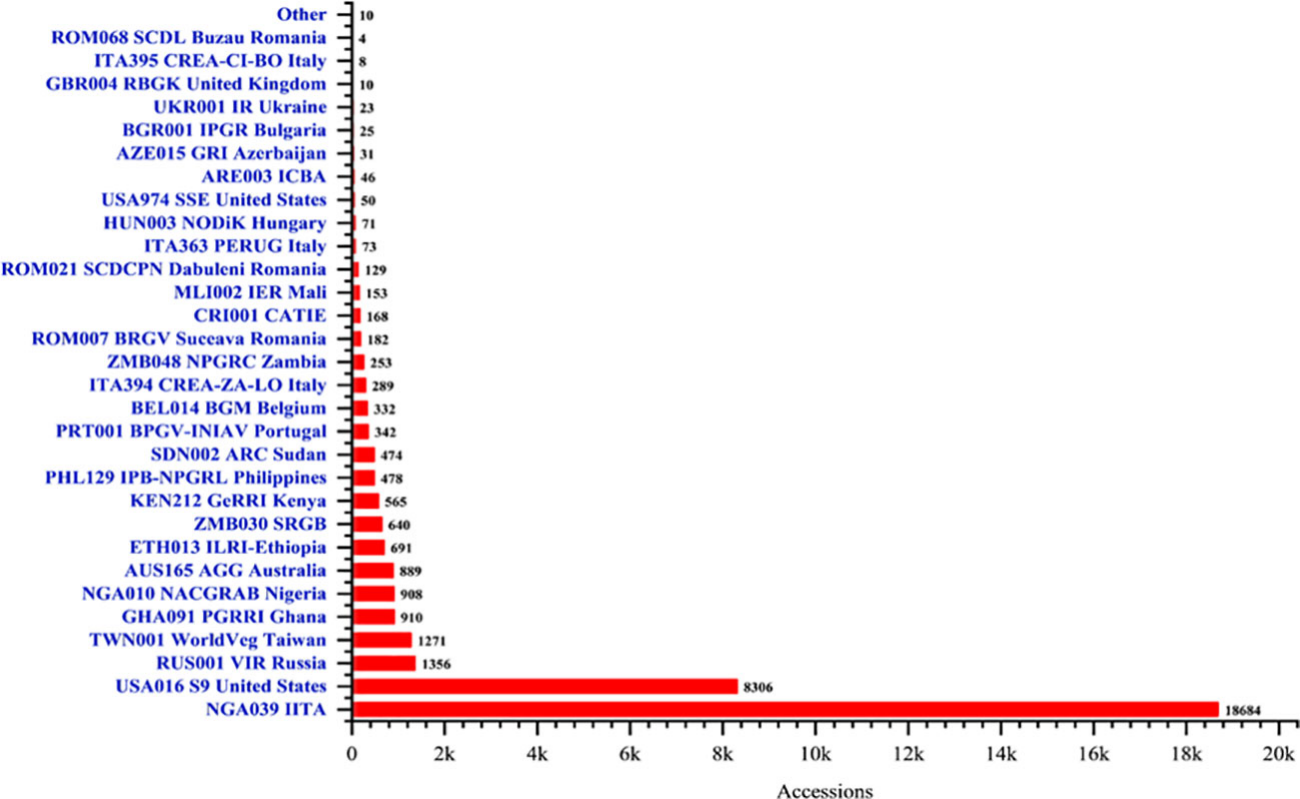
Global cowpea germplasm conservation status, showing various institutions dedicated to the preservation of cowpea germplasm. Generally, Nigeria (IITA and NACGRAB), United States, Russia (VIR), Taiwan (WorldVeg), Ghana (PGRRI), and Australia (AGG) conserves the largest proportion of the global cowpea genetic resources.

The worldwide collection and conservation of germplasm are vital components of global efforts to enhance agricultural sustainability and food security. The conservation efforts in safeguarding cowpea genetic diversity warrants the resilience of the crop to environmental challenges and contribute to breeding efforts for improved varieties. The collection of cowpea germplasm since decades ago has been a collaborative endeavor involving agricultural research institutions, gene banks, and international organizations worldwide (Boukar et al., 2019a). These efforts aim to preserve cowpea genetic diversity. Major germplasm collections exist in the domestic Genebanks of countries with significant cowpea cultivation, and academic institutions, as well as in international Genebanks and research centers (genesys-pgr.org/c/cowpea) (Nair et al., 2023) (**Figure 3**). The IITA Genebank remains the foremost repository for the world’s most diverse cowpea germplasm, safeguarding more than 16,000 accessions, which include some 2000 wild relatives (https://my.iita.org/accession2/ accessed on 2024/07/19). In the United States, the University of California, Riverside, and the USDA repository in Griffins, Georgia, together conserve around 12,000 accessions (Herniter et al., 2019; Muñoz-Amatriaín et al., 2017). Other Genebanks, including the National Bureau of Plant Genetic Resources (NBPGR) in India (Horn et al., 2022; Raizada et al., 2023), Banco Português de Germplasm Vegetal (INIAV) in Portugal (Moreira et al., 2023) and the National Agrobiodiversity Center Genebank in the Republic of Korea, also significantly contributed to the global conservation of cowpea genetic resources (Seo et al., 2020). Around 4,301 cowpea accessions are conserved *ex situ* in European national gene banks (Lazaridi and Bebeli, 2023). This indicates the global efforts in cowpea conservation. Despite the collecting and conservation efforts, challenges exist, such as inadequate infrastructure and insufficient capacity for long-term storage and maintenance of germplasm collections, especially, domestic Genebanks in Africa where species of the crop are highly endemic. For instance, Ghana’s Genebank at the Plant Genetic Resources Research Institute of the Council for Scientific and Industrial Research harbors a substantial amount of cowpea germplasm and capable of increasing its collecting capacity, but lacks facilities for long term sustainable conservation of its current accessions. The predicted threats of future climate change and the unabated level of habitat loss calls for increased effort in cowpea germplasm conservation, where climate-resilient traits, such as drought tolerance, heat resistance, and pest and disease resistance may be obtained.

## 6 Breeding advances of cowpea

### 6.1 International breeding initiative

Cowpea breeding has a rich history, marked by concerted efforts to improve on key traits such as yield potential, biotic and abiotic stress tolerance. In cowpea, early improvement strategies focused on the selection of landraces with desirable agronomic characteristics. It was not until the mid-20th century, when formal breeding initiatives were pioneered and gained momentum, particularly countries in Africa where cowpea is a major staple (Singh, 2016; Boukar et al., 2019b). The IITA continue to play a crucial global mandate role in the development of new cowpea varieties (Madina et al., 2024). Globally, cowpea breeding has been driven by international collaborations. The Collaborative Research Support Program (CRSP) in the USA, initiated in the 1980s provided significant support in promoting cowpea research and breeding efforts across Africa and Asia (Hall et al., 2003). Since then, scientists at the IITA have developed diverse varieties with desirable characteristics such as resistance to pests, diseases, weeds and abiotic stresses (Horn and Shimelis, 2020). More information on improved cowpea varieties developed at IITA can be found in previous published works (Singh, 2016; Boukar et al., 2019a, Boukar et al., 2019b). Building on this foundation, recent multinational research initiatives are now targeting more fundamental plant traits to strategically enhance cowpea breeding. Notable among these are the Realizing Increased Photosynthetic Efficiency (RIPE) and ENSA (Enabling Nutrient Symbioses in Agriculture) programs, which are contributing to the next generation of cowpea improvement by targeting foundational plant traits beyond conventional breeding targets. The RIPE project, led by the University of Illinois and supported by the Bill & Melinda Gates Foundation, seeks to boost photosynthetic efficiency in crops such as cowpea, with the goal of increasing yield under conditions of climatic stress (Digrado et al., 2020; https://ripe.illinois.edu/press/2022, Accessed on 2025/05/29). Improved photosynthetic efficiency directly supports cowpea breeding priorities related to yield enhancement and adaptation to climate variability (Taylor et al., 2022). Complementing this effort, the ENSA project focuses on engineering improved nitrogen-fixing symbioses in legumes like cowpea, thereby reducing the need for synthetic fertilizers and enhancing soil fertility (Oldroyd and Dixon, 2014; https://www.ensa.ac.uk, Accessed on 2025/05/29). These cutting-edge programs align with the long-term breeding objectives of cowpea, including improved photosynthetic capacity, greater resilience to abiotic stress, and sustainable nutrient use. When integrated with traditional breeding and modern molecular approaches, the outcomes of RIPE and ENSA are expected to significantly accelerate genetic gains in cowpea, by introducing advanced traits that support climate resilience, resource efficiency, and sustainable agricultural intensification. This hold the potential to ultimately contribute to climate-smart and economically viable farming systems, particularly for smallholder farmers in Africa and Asia.

Over the past few years, significant progress has been made in applying biotechnology tools to genetically improve cowpea, particularly in addressing key production and post-harvest constraints. Among the most critical achievements is the development of cowpea varieties resistant to Maruca vitrata, a lepidopteran pest responsible for yield losses of over 80% in affected areas (Nboyine et al., 2024a; Addae et al., 2020). A landmark development in this regard was the successful deployment of the Pod Borer Resistant (PBR) cowpea. This biotechnology innovation was spearheaded by the African Agricultural Technology Foundation (AATF), which played a central role in coordinating research, development, and commercialization efforts (https://www.aatf-africa.org/pod-borer-resistant-maruca-pbr-cowpea, Accessed on 2025/05/29). In 2019, Nigeria approved and released Sampea 20-T, the first genetically modified PBR cowpea variety in Africa (Amedu et al., 2023). This milestone was the result of a multi-institutional collaboration involving the Institute for Agricultural Research (IAR), Ahmadu Bello University, CSIRO (Australia), the National Biotechnology Development Agency (NABDA), Agricultural Research Council of Nigeria (ARCN), Danforth Plant Science Center, and Bayer CropScience. The project received sustained funding from USAID and broad support from local stakeholders, including farmer associations and seed companies. Sampea 20-T was developed, using advanced biotechnological methods to introduce a resistant gene expressing the Cry1Ab insecticidal protein from *Bacillus thuringiensis* (Bt) (Nboyine et al., 2024a; Amedu et al., 2023). This innovation provides effective protection against Maruca vitrata and marks a significant milestone in Africa’s biotechnology crop landscape. Its release has led to measurable benefits: in Nigeria, Sampea 20-T has been cultivated on over 10,000 hectares, resulting in up to 80% reduction in insecticide use, lower production costs, enhanced environmental outcomes, and improved farmer profits. Additionally, improved grain quality has boosted market acceptance. Following this, Ghana’s Council for Scientific and Industrial Research, the Savannah Agricultural Research Institute (CSIR-SARI) pursued a similar innovation. In 2024, after nearly a decade of confined and on-farm trials, Ghana released Songotra-T, a cowpea variety developed with comparable resistance traits (Nboyine et al., 2024b; https://csir-sari.org/2024/07/25/ghana-launches-first-home-grown-gm-crop). The development of Songotra-T was made possible through transboundary collaboration with several external partners. This variety showcases the regional spillover benefits of biotechnology and cross-country research efforts. Both Sampea 20-T and Songotra-T combine robust resistance to Maruca vitrata with desirable agronomic characteristics, such as high yield potential, drought tolerance, and good grain quality—traits critical to smallholder farmer adoption (Nboyine et al., 2024a; Amedu et al., 2023).

Another major challenge limiting cowpea production is post-harvest grain lost caused by the cowpea bruchid (*Callosobruchus maculatus*), a highly destructive storage pest. Under traditional storage conditions, especially in tropical regions, *C. maculatus* infestation can result in up to 100% grain loss within six months, posing serious threats to food security, and farmer livelihoods (Nboyine et al., 2024b). In response to this critical constraint, recent collaborative research initiatives have made significant progress using transgenic approaches. A notable A landmark study emerged from a multi-institutional collaborative effort, involving the Council for Scientific and Industrial Research–Savanna Agricultural Research Institute (CSIR–SARI, Ghana), Institute for Agricultural Research at Ahmadu Bello University (Nigeria), the Commonwealth Scientific and Industrial Research Organization (CSIRO, Australia), and the Institute for International Crop Improvement at the Donald Danforth Plant Science Center (USA). Together, these institutions developed a genetically modified (GM) cowpea event (CSI-32), which expresses the *Phaseolus vulgaris* (common bean) alpha-amylase inhibitor 1 (αAI-1) protein specifically in the seed tissue (Nboyine et al., 2024a, Nboyine et al., 2024b). The αAI-1 protein functions by interfering with starch digestion in the larvae of *C. maculatus*, thereby effectively halting their development and emergence. Field trials conducted in Ghana and Nigeria in 2023 compared CSI-32 with its non-transformed parental line (IT86D-1010) as well as with commercially released varieties including SAMPEA 7, SAMPEA 20-T, and IT13K-1070-2. The results were promising, with CSI-32, demonstrating agronomic trait equivalence with the check varieties in terms of growth, yield, and phenotypic traits, while demonstrating complete suppression of bruchid emergence during a four-month post-harvest storage period. These findings confirm earlier work (Solleti et al., 2008), which demonstrated the potential of αAI-1 for bruchid resistance in transgenic cowpea under laboratory conditions. These transgenic lines present a sustainable and highly effective strategy for mitigating post-harvest losses, reducing reliance on chemical insecticides, and enhancing the market value of stored cowpea grain for smallholder farmers. Overall, this biotechnological advancement represents a critical milestone for cowpea improvement programs. It provides a durable genetic solution to one of the crop’s most intractable storage challenges and highlights the growing role of genome-based and transgenic approaches in improving legume resilience and food security in sub-Saharan Africa.

### 6.2 Cowpea research and breeding development in Africa

It is over five decades now (around 1950s and 1960s) when the cowpea research initiatives unfolded in parts of Africa (Carvalho et al., 2017), laying the groundwork for more systematic breeding programs that followed. The involvement of international organizations like Canada’s International Development Research Centre (IDRC) boosted research across multiple African countries from the late 1970s onwards (Boukar et al., 2019a). From germplasm collection, through experimental screening and selection of desirable cowpea resources, currently, extensive research and breeding of cowpea spans across several countries and institutions in the region. In Africa, the IITA has played a central role in driving cowpea improvement through advanced breeding programs, capacity building, and technology dissemination. Cowpea breeding goals in Africa targets releasing varieties, characterized by improved yield, desirable maturity time, and resistance to environmental stresses. For instance, the IITA has developed and released several varieties of cowpea that are not only high yielding but which also exhibit resistance to various kinds of biotic and abiotic stresses, as well as quality attributes such as consumer- and market-preferred seed color (Ongom et al., 2024). Recent projects, such as the Accelerated Varietal Improvement and Seed Delivery of Legumes and Cereals in Africa (AVISA) initiative, funded by the Bill & Melinda Gates Foundation have further strengthened the pipeline for breeding and seed system development (https://www.iita.org/news-item/avisa-project-showcases-improved-crop-varieties-at-farmers-field-day/). Through this project, new cowpea varieties with desirable traits have been introduced to farmers. For instance, FUAMPEA1 and SAMPEA14 which have a characteristic early maturity, high pod load and yield, and resistance to parasitic weed (*Striga gesnerioides*) hold the potential to boost cowpea grain yield. In addition to works carried out by IITA, the Federal Department of Agricultural Research, the Institute for Agricultural Research and Training (IAR&T), located in Ibadan (Nigeria), and the Centre National de Recherches Agronomiques (CNRA) in Senegal are noted for their forefront of cowpea improvement (Boukar et al., 2019a). Targeting improved desired seed quality attributes, the Ghana Cowpea Product Design Team from CSIR-Savannah Agricultural Research Institute and the Cowpea Breeding Unit of IITA have aimed to develop lines tailored for diverse market demands in Ghana’s Northern climate (https://csir-sari.org/#features). These efforts include creating boiled grain and homemade flour varieties in white and brown types, alongside dual-purpose varieties for both grain and fodder applications. Nevertheless, significant challenges remain including diseases and pests, parasitic weeds (Omomowo and Babalola, 2021; Togola et al., 2023). There is a need for effective and efficient distribution of certified seeds of improved cowpea varieties to farmers. Efforts are also required to promote technologies such as marker-assisted selection and genomic selection in cowpea breeding. For some institutions, the application of these tools is still constrained by inadequate laboratory infrastructure, funding limitations, and limited trained personnel (Muñoz-Amatriaín et al., 2017; Togola et al., 2023). Furthermore, regulatory frameworks for the adoption of genetically improved crops, including biotech cowpea, remain inconsistent across the continent, creating delays and uncertainties in the release and commercialization of innovations. Strengthening public-private partnerships, enhancing farmer education, investing in biotechnology infrastructure, and harmonizing biosafety regulations are critical steps needed to overcome these hurdles and maximize the impact of IITA-led advancements on cowpea production in Africa.

### 6.3 Cowpea research and breeding development in the Americas

In the United States, cowpea is grown for various purposes including as a vegetable, for dry beans and as soil cover crop, which enhances the soil, particularly in organic farming systems. Research and breeding development prioritize end-use–specific traits, such as grain yield and quality, along with resistance to biotic and abiotic stresses (Osipitan et al., 2021). Various research institutions have played key roles since the 1980s in cowpea research in the USA, including the United States Department of Agriculture (USDA) and universities like University of Arkansas, University of California, Riverside, University of Georgia, Purdue university, Louisiana State University, Mississippi State University, Texas A&M, University, Auburn University, and Clemson University (Boukar et al., 2019b). They collaborate closely with farmers and industry stakeholders to identify traits that can increase yields and improve marketability. Key objectives include developing varieties with enhanced yield, resilience to diverse environmental conditions such as drought tolerance, and pest and disease resistance, as well as improving nutritional qualities for the growing demand for sustainable and nutritious food sources (Osipitan et al., 2021). Genetic studies and breeding programs also aim to diversify cowpea cultivars suited to different regions across the country, ensuring robust production and economic viability for farmers. In the Southern America, the Brazilian Agricultural Research Corporation (EMBRAPA), leads efforts in genetic improvement and adaptation studies to enhance the productivity of cowpea under varying climate conditions (Barbosa et al., 2016). EMBRAPA has collaborated with IITA, in initiating early cowpea variety improvement programs (Ongom et al., 2024). Breeding programs have also considered developing cultivars that are resistant to local pests and diseases, as well as tolerance to drought and soil salinity. Research also aims to elevate the nutritional quality of cowpea through biofortification initiatives, addressing regional dietary deficiencies. Significant research on cowpea is also carried out in Other South American countries like Colombia, Venezuela, Panama, Trinidad, Nicaragua, Jamaica and Guyana (Boukar et al., 2019b).

### 6.4 Cowpea research and breeding development in Asia

Cowpea research and breeding are integral to strengthening food security and promoting sustainable agriculture in Asia. Efforts are geared towards developing high-yielding varieties that are resilient to pests, diseases, and adverse environmental conditions such as heat and drought. Asian countries, including India, Bangladesh, Myanmar, China, Indonesia, Nepal, Pakistan, the Philippines, Sri Lanka and Thailand are known to have contributed significantly to cowpea cultivar development (Boukar et al., 2019b; Pathak and Biologist, 2023). For the Republic of Korea, cowpea is still a minor crop in terms of production and consumption (Seo et al., 2020), with research aimed at developing varieties suitable for the temperate climatic condition, early maturity for short growing seasons that are adapted to combine harvesting (Kim et al., 2018; Choi et al., 2022, Choi et al., 2023). Breeding programs may also target traits such as disease resistance, and high yield potential to optimize production in limited agricultural land. In their recent study, Seo et al. mentioned that only four cowpea varieties have been developed in South Korea, and emphasized the need to exploit the available genetic resources for developing new cultivars (Seo et al., 2020). “Seonhyeon” “Okdang,” Okhyun”, and “Jang-alchan”, are among cowpea varieties developed in the Republic of Korea (Kim et al., 2018, Kim et al., 2019), with the Jeollanamdo Agricultural Research and Extension Services (JARES) at the center in developing these verities (Choi et al., 2022, Choi et al., 2023). Seonhyeon, developed in 2017, features an erect growth habit, black seeds, and a higher anthocyanin content with a yield of 2.26 tons/ha. Using the pure line isolation method, Okdang was developed in 2013 by the JARES, which exhibits intermediate growth habit, strong resistance to lodging, and erectness, with heavier seeds that yield 1.85 tons/ha (Kim et al., 2018). In India, cowpea breeding targets include developing varieties with improved traits suitable for use as vegetable; such as varieties bearing stringless tender pods (Aghora et al., 2024a). Other desirable traits targeted include improved pod quality characteristics such as soft, long, pulpy and parchment free pods in vegetable cowpea. For grain cowpea, breeding targets determinate growth habit, and earliness in flowering and fruiting (Aghora et al., 2024a).

### 6.5 Cowpea research and breeding development in Europe

While European climate is less extreme in terms of heat and drought compared to the tropical regions where cowpea is typically grown, the crop still faces significant abiotic and biotic stresses, especially in the Southern Europe (Lazaridi and Bebeli, 2023). These challenges, along with various yield-limiting factors, hinder the full production potential of the crop in the region, necessitating strategic breeding efforts. Breeding for resistance to environmental stresses is a crucial objective to address the various challenges affecting seed and pod production in cowpea cultivation areas. For instance, developing drought-tolerant cultivars is a key breeding goal in cowpea breeding programs that can facilitate stable production under limited water conditions (Carvalho et al., 2017). Additionally, enhancing yield is a key objective to maximize the potential of the crop in temperate climates, despite the stresses it encounters (Lazaridi and Bebeli, 2023). In Europe, institutions and universities in countries like Italy engage in genetic improvement programs to develop cowpea varieties suited for European conditions. Currently, about 16 cowpea varieties are registered in the national catalogues of European countries (Draghici et al., 2018; Rubiales et al., 2021; Nunes et al., 2022).

## 7 The potential of OMICs technologies in genetic improvement of cowpea

The application of OMICs technologies in cowpea genetic improvement has become increasingly significant in addressing key challenges in agriculture, such as climate change, pests, and diseases. OMICs, including genomics, transcriptomics, proteomics, and metabolomics provide a comprehensive approach in understanding the genetic, molecular, and biochemical mechanisms that underlie the adaptability and productivity of crops. Recent studies have demonstrated the potential of these technologies in genetic improvement efforts for cowpea.

### 7.1 Advances in cowpea genomics research and breeding

The integration of genomic tools into cowpea breeding programs holds immense potential for accelerating the development of improved varieties. Genomics involves the comprehensive analysis of the cowpea genome, enabling the identification of genes and markers associated with desirable traits such as drought tolerance, pest resistance, and enhanced nutritional content. High-throughput sequencing technologies, such as next-generation sequencing (NGS), facilitate the generation of reference genomes and genome-wide association studies (GWAS). These approaches allow breeders to pinpoint loci controlling key agronomic traits, thereby streamlining marker-assisted selection (MAS) and genomic selection (GS) processes (Boukar et al., 2019a; Jha et al., 2020). Lonardi et al. provided an in-depth analysis of the cowpea genome, identifying critical genes associated with biotic and abiotic stress responses (Lonardi et al., 2019). These genomic insights have accelerated breeding programs aimed at developing climate-resilient cowpea varieties. The completion of the cowpea genome sequence (Lonardi et al., 2019) has further improved the resolution of genetic studies, paving the way for more targeted and efficient breeding strategies. Comparative genomics can be employed to identify syntenic regions across related legume species, aiding in the discovery of conserved genes that can be leveraged for breeding purposes (Lonardi et al., 2019). In their research, Boukar et al. emphasized the importance of genomics in cowpea breeding, and that genomic tools are pivotal for identifying genes related to key traits such as drought tolerance, disease resistance, and nutrient content, which are among the most important concern in cowpea production in many parts of Africa (Boukar et al., 2019a). Developing a robust genomic database for cowpea germplasm resources will further enable breeders to harness the genetic diversity of the crop more effectively (Muñoz-Amatriaín et al., 2017). By combining genomic data with phenotypic and environmental data, breeders can predict the performance of varieties under varying conditions, ensuring sustainable production (Aghora et al., 2024a). Moreover, the incorporation of genomic tools into breeding programs will expedite the identification of cowpea varieties with improved traits tailored to specific regional challenges, from pest management to enhanced nutritional quality (Boukar et al., 2016). Therefore, prioritizing genomic research in cowpea breeding programs is essential for meeting future food security demands. Genome editing technologies, particularly CRISPR-Cas9, offer precise modifications to target genes for improved traits, such as enhancing nitrogen fixation efficiency or reducing anti-nutritional factors (Jha et al., 2020). Currently, efficient transformation is a bottleneck in functional genomics and trait introduction in cowpea. Recent advances have significantly improved Agrobacterium-mediated transformation efficiency. Specifically, Popelka et al. (2023) and Che et al. (2024) reported improved cowpea regeneration and transformation systems, including the use of embryonic axis explants, enhanced virulence induction media, and genotype-specific protocols. These advances have enabled effective transgene insertion and expression in elite African cowpea cultivars, opening up new possibilities for trait-specific improvement, including disease resistance, nutritional improvement, and climate tolerance.

### 7.2 Advances in transcriptomics in cowpea breeding

The application of transcriptomics profiling, or RNA sequencing (RNA-seq) has provided insights into gene expression dynamics underlying abiotic and biotic stresses in cowpea. Spriggs et al. (2018) revealed in their study the transcriptomic responses that are relevant to mechanisms of drought tolerance, and that highly regulated genes govern stomatal closure, osmotic adjustment, and antioxidant defense processes. Similarly, transcriptomic analysis after aphid infection identified several differentially expressed genes (DEGs), e.g., pathogenesis-related proteins and defense signal pathways (MacWilliams et al., 2023). Such research facilitates the selection of candidate genes suitable for genetic modification or specific breeding interventions. In a previous study, the value of transcriptomics in understanding gene expression changes in response to environmental stresses such as heat and drought was highlighted (Diouf, 2011; da Silva Matos et al., 2022). This research revealed that specific transcription factors are activated under stress conditions, which could be harnessed in breeding programs to develop more resilient cowpea varieties. This highlights the potential of transcriptomics in identifying gene networks that can be manipulated to improve stress tolerance in cowpea. A previous study identified reference genes for transcriptional profiling in cowpea under abiotic stress conditions (Amorim et al., 2018). They highlighted the potential for qPCR-based studies to monitor stress responses. In a similar study, transcriptomic analyses of kinome of cowpea was performed, and revealed the involvement of key kinases in biotic and abiotic stress responses, which could serve as targets for breeding more resilient varieties (Ferreira-Neto et al., 2021). Their study noted that the kinome plays a critical role in stress signal transduction, which could be crucial for developing cowpea varieties resistant to drought and pests. A noteworthy area of focus has been the response of cowpea to salt stress. A transcriptome analysis during the early vegetative stage under salt stress was previously reported, with differentially expressed genes (DEGs) identified to be associated with stress tolerance (Kang et al., 2023). Their findings highlighted genes involved in ion transport, osmoregulation, and antioxidant defense, providing valuable insights into the genetic mechanisms that could be leveraged for breeding salt-tolerant cowpea varieties. While these studies provide valuable insights into gene expression under specific stress conditions, there is still a gap in understanding how these genes behave across different cowpea varieties and environmental contexts. As noted in a recent study, there is a need for further in-depth research that explores gene regulatory networks and differential gene expression across diverse genotypes and varying environmental factors (Krylova et al., 2024). Understanding these patterns would aid in developing more robust cowpea varieties that can thrive under fluctuating environmental conditions, especially in the face of climate change. Furthermore, integrated approaches, such as combining transcriptomics with metabolomics, have shown promise in understanding complex traits like anthocyanin accumulation. Li et al. conducted an integrated analysis of metabolomics and transcriptomics in cowpea pods, and reported the molecular mechanisms behind flavonoid accumulation (Li et al., 2020). Their study demonstrated how transcriptomic data could complement metabolic profiling to identify key genes regulating important nutritional traits. The recently published *Vigna unguiculata* transcriptome atlas (Yao et al., 2023) provides a high-resolution spatial and temporal gene expression data set for a range of tissues and developmental stages. This atlas is a valuable resource for gene discovery, promoter mining, and understanding tissue-specific regulation of key agronomic traits. Through the integration of such types of transcriptomic data into molecular breeding pipelines, researchers can prioritize candidate genes for drought tolerance, pest resistance, and seed quality improvement, as well as support the development of gene expression markers.

### 7.3 Advances in proteomics in cowpea breeding

Cowpea proteomics is comparatively less developed but does possess significant potential in unraveling protein-level regulatory mechanisms of nutritional quality and stress adaptation. In a proteomic study, drought-responsive proteins such as heat shock proteins (HSPs), antioxidant enzymes, and regulators of metabolism were identified as crucial proteins involved in protecting cowpea cells from drought-induced oxidative damage (Agbicodo et al., 2009). They were also found to be involved in stabilizing cellular structures, and maintaining metabolic balance under water-deficit conditions. Some recent research also employed high-level mass spectrometry (MS)-based proteomics to profile seed storage proteins and bioactive peptides in cowpea, and determined their likely nutritional and health benefits (Vasconcelos et al., 2010). A study by Ribeiro et al. focused on proteomic analysis to investigate the response of cowpea to combined biotic and abiotic stresses, where key proteins involved in stress responses were identified (Ribeiro et al., 2023). This proteomic profiling can facilitate the development of biomarkers for selecting stress-tolerant genotypes, and thus enable a rapid and more precise breeding decision. A study indicated that differential proteomic analysis can identify proteins related to water stress tolerance, a critical trait for cowpea cultivation in drought-prone regions (Lima et al., 2019). As consumer demand for high-quality, nutritious food increases, proteomics can play a significant role in breeding cowpea varieties with improved grain quality. By enhancing our understanding of the proteomic landscape of cowpea, breeding programs can better address the dual goals of food security and nutritional security. Prioritizing proteomics research will ensure cowpea remains a sustainable and versatile crop for future generations (Raizada et al., 2023). The combination of genomic and proteomic strategies promises to enhance the productivity, resilience, and nutritional value of the crop, making it a key player in meeting the world’s growing food security challenges. Functional proteomics provides opportunities to explore protein-protein interactions, offering insights into complex regulatory networks controlling key traits (Jha et al., 2022). This approach helps to better understand how proteins interact to regulate critical processes such as flowering, seed formation, and stress responses. Future development in cowpea proteomics may consider determining functional protein networks that are vital for cowpea stress adaptation and utilization.

### 7.4 Advances in metabolomics in cowpea breeding

Metabolomics has provided valuable insights into the biochemical pathways associated with important traits such as drought resistance and nutritional quality. For instance, the study by Goufo et al. demonstrated how metabolomics can be used to understand osmoprotection mechanisms that enhance drought tolerance in cowpea, which further illustrates the role of OMICs in improving crop resilience (Goufo et al., 2017). In their comprehensive review, Diouf noted that OMICs technologies are not only beneficial for understanding stress tolerance but also for improving the nutritional quality of cowpea (Diouf, 2011). Metabolomics is a powerful tool to profile metabolic constituents, in particular, to identify those metabolites associated with stress tolerance and nutritional value in cowpea. Metabolite profiling using techniques like ultra-performance liquid chromatography-mass spectrometry (UPLC-MS) and nuclear magnetic resonance (NMR) has identified secondary metabolites, e.g., flavonoids, saponins, and phenolics, involved in drought tolerance and pest resistance (Goufo et al., 2017). Metabolomic research has also been critical in the identification of metabolites involved in nutritional quality, e.g., essential amino acids and vitamins, thereby guiding breeding programs for biofortification purposes (Dakora and Belane, 2019). In a previous study, the roles of plant secondary metabolites from cowpea floral structures were explored in relation to resistance against flower bud thrips (Alabi et al., 2011). The results revealed that specific flavonoids and phenolic compounds in the flowers contributed significantly to pest resistance mechanisms. These findings highlight the importance of floral metabolite composition in developing pest-resistant cowpea varieties. In a study focusing on cowpea lines, it was found that secondary metabolites such as tannins, flavonoids, and saponins mediated resistance to flower thrips (*Megalurothrips sjostedti* Trybom), which further emphasized the role of biochemical defenses in cowpea breeding strategies targeting pest resilience (Oladejo et al., 2025). Genotype × environment interaction was shown to influence secondary metabolite accumulation in cowpea under thrips infestation, as reported in another study (Gitonga et al., 2022). The results demonstrated that both genetic background and environmental conditions shape the metabolite profile, which in turn affects the plant’s capacity to withstand biotic stresses, suggesting that metabolomics-assisted selection should consider environmental variability. Seed coat metabolite profiling of cowpea accessions from Ghana using UPLC-PDA-QTOF-MS and chemometrics identified a diverse range of compounds, including phenolic acids and flavonoid derivatives (Tsamo et al., 2020). This study underscored the nutritional and protective roles of seed coat metabolites, offering insights for both breeding programs and food science applications aimed at enhancing cowpea’s functional properties. In another study, the metabolite profiles of local cowpeas from Southwest Maluku, Indonesia, demonstrated considerable variation in composition among accessions (Karuwal et al., 2023). Key metabolites related to flavor, nutrition, and potential health benefits were characterized, emphasizing the value of metabolomics in uncovering unique genetic resources for crop improvement and local food security. These advances illustrate the growing role of metabolomics in enhancing our understanding of the biochemical diversity, stress tolerance mechanisms, and nutritional potential of cowpea, and ultimately support the potential of developing superior cowpea cultivars for diverse agroecological contexts.

### 7.5 Integrated OMICs technologies in cowpea breeding

Integrating genomics, proteomics, and metabolomics has enabled the identification of genes and metabolic pathways involved in the biosynthesis of essential nutrients, which could be enhanced for biofortification efforts (Zhang et al., 2022). Such efforts are crucial in addressing malnutrition and improving the nutritional security of populations dependent on cowpea as a staple food. Integration of various OMICs tools provides unparalleled opportunities to holistically understand cowpea plant biology, requisite for designing breeding programs. Integrative research over the last few years has provided detailed insights into the complex trait architecture nature of cowpea, particularly under conditions of environmental stress, enabling breeders to develop more robust and resistant varieties (Spriggs et al., 2018; Boukar et al., 2019a). Increased emphasis on multi-omics integration will most likely lead to revolutionary improvement of agronomic performance, stress resistance, and nutritional content of cowpea varieties. Thakur et al. discussed the role of OMICs in enhancing fodder cowpea, noting the importance of combining genomic, proteomic, and metabolomic data to identify key genes involved in stress responses (Thakur et al., 2024). Their study emphasized how integrated OMICs approaches could lead to a more comprehensive understanding of gene functions, enabling the development of cowpea varieties with improved resilience to both biotic and abiotic stresses. Similarly, another recent study highlighted the use of OMICs technologies to enhance abiotic stress tolerance in legumes, including cowpea (Ali et al., 2022). Their findings revealed that integrating genomic and metabolomic data could provide insights into the biochemical pathways underlying stress tolerance, thereby facilitating targeted breeding for stress-resilient varieties. Further, the application of gene-editing technologies, such as CRISPR-Cas9 now has opened new avenues for precise genetic manipulation in cowpea. Recent work by Ji et al. demonstrated the successful use of CRISPR-Cas9 to edit genes in cowpea, and the potential for developing varieties with improved traits such as pest resistance and enhanced nutrient content (Ji et al., 2019). This genome-editing approach, when combined with OMICs data, holds the promise of accelerating the development of improved cowpea varieties with desired traits.

## 8 Conclusion and future perspective

Cowpea holds immense potential for addressing food security challenges, especially in regions vulnerable to climate change. As a nutritious, adaptable, and resource-efficient crop, its importance in the global food system is expected to grow, especially given continued advancements in germplasm exploration, breeding and production expansion. With Africa as the largest producer and consumer of cowpea, the adoption of improved varieties, research and farmer training as well as modern agricultural techniques such as mechanized farming will be crucial for increasing productivity. Cowpea production in regions like Asia and parts of the Americas and Europe can diversify food sources and enhance nutritional security. Strengthening international breeding collaborations will be essential to sharing knowledge and developing region-specific varieties. The International Institute of Tropical Agriculture will continue to play a pivotal role in coordinating these efforts. Future breeding priorities should focus on improving yield, abiotic and biotic stress resistance, and nutritional quality. Developing varieties with enhanced adaptability to local environmental challenges will ensure the sustainability of cowpea as a food source worldwide. Improved grain quality, such as nutritional value (higher protein content and better amino acid profiles), seed size, and cooking time will increase marketability and consumer preference. Cowpea plays a key role in improving soil health through nitrogen fixation. Thus, breeding programs may target enhancing this trait, such as breeding super nodulation varieties for promoting sustainable agriculture. With advances in genomics technology, application of molecular tools like genome editing holds great potential in future cowpea breeding. Collaborative research will be valuable in breeding resilient cowpea varieties, while exploring underutilized cowpea germplasm and increasing farmer participatory breeding initiatives to promote easy and wider adoption. As climate change continues to pose challenges to agriculture, the application of OMICs tools will also be crucial in developing climate-resilient cowpea varieties. The use of OMICs technologies in cowpea research has significant potential for advancing breeding strategies and improving cowpea production. Efforts should also be geared towards addressing socio-economic barriers, including market access and policy support to maximize potential contribution of cowpea to global food and nutritional security. Overall, the future of cowpea as a global food security crop will very much rely on the availability and accessibility to genetic resources, and sustained investment in research and breeding programs.
